# A Clinical and Practical Treatise on the Diseases of Children

**Published:** 1845-10

**Authors:** 


### I. TRAITÉ CLINIQUE ET PRATIQUE DES MALADIES DES ENFANS. Par MM. *Rilliet* et *Barthez*. 8vo. pp. 2370. Paris, 1843.

A Clinical and Practical Treatise on the Diseases of Children. By MM. *Rilliet* and *Barthez*.

### II. MANUEL PRATIQUE DES MALADIES DES NOUVEAUX-NÉS ET DES ENFANS A LA MAMMELLE. Par *E. Bouchut*. Small 8vo. pp. 610. Paris, 1845.

A Practical Manual of the Diseases of New-born Infants and Children at the Breast. By *E. Bouchut*.

[Second Notice.]

OUR readers will recollect that MM. Rilliet and Barthez arrange the diseases treated of in their important work in eight classes, Inflammations, Dropsies, Hæmorrhages, Gangrenes, Neuroses, Fevers, Tuberculizations and Entozoaires. The four first of these we have already examined; and now proceed to analyse the most important facts relating to those which remain.

### CLASS V.—NEUROSES.

The nervous affections most commonly observed in childhood are denoted especially by a perversion or abolition of muscular contractility, and those here treated of are mostly of a convulsive character. They are *Pertussis, Spasm of the Glottis, Convulsions, Chorea, Contractions,* and *Essential Paralysis.* All these diseases, in their uncomplicated state, agree in being devoid of fever and of pain; some of them being intermittent, and others continued, but with exacerbations. Complications with other diseases, or with each other, are however, very frequent, and much modify the progress and results of these affections.

### *Pertussis.*

This disease is excellently described in both the French works under review, but, without going into the detail, we may extract a few observations from that of MM. Rilliet and Barthez.

*Auscultation.*—" The complications are for the most part seated in the lungs, and as the bronchitis and pneumonia are recognised by the rapidity of pulse and respiration, and the auscultatory signs, it is quite necessary to examine the little patients daily in the *interval of the paroxysms.* In fact, if the ear be applied at the instant of a paroxysm, independently of the material obstacles offered by the resistance of the child, no respiratory murmur is heard, and we can only perceive the anormal resonance of the cough. If we auscult during the whooping inspiration which succeeds the interrupted expirations, the respiratory murmur is not heard either, the air not penetrating beyond the larger divisions of the bronchi. It is then quite necessary to examine the child at a period somewhat remote from the fit of coughing; care must be taken also to count the pulse and respirations some time prior to or subsequent to a paroxysm, for we have almost always observed an acceleration of the pulse and breathing to occur a little while prior to the paroxysm, and to persist for some time after its termination."

*Complications.*—These may be arranged under three heads. 1. Those which belong to the nature of the disease, as convulsions and spasm of the glottis. 2. Those which depend upon its locality, as bronchitis, pneumonia, hæmorrhages, and tubercles. 3. Those which are mere coincidences as pleurisy, meningitis, eruptive fevers, &c. &c. *Convulsions* are by no means a rare complication, especially in young children, and, occurring during the paroxysmal stages of the affection, add much to the gravity of the disease. *Bronchitis,* as all know, is a very common complication, being attended with dilatation of even the smallest bronchi. *Lobular pneumonia* also is a frequent complication, often co-existing with the bronchitis. MM. R. and B. do not regard *Emphysema* as one of the complications, never having found it, except when the patient has succumbed to pneumonia or bronchitis, and not always then; and never so considerable as when these inflammatory affections existed without pertussis. Indeed they regard the repeated expirations, followed by the prolonged spasmodic inspiration of hooping-cough, expelling the air, and then opposing its free re-admission, as unfavourable to the production of emphysema; while the dilatation of the bronchi observed results solely from their inflamed condition. M. Bouchut, however, states that emphysema is frequently met with. Of *Hydrocephalus* and *Anasarca*, stated by Frank and Lombard as complications, the authors speak with just scepticism. *Epistaxis* is not uncommon, but is a mere mechanical effect of the violence of the paroxysm; and the *Hæmatemesis*, reported by some authors, has resulted from the regurgitation of the blood from the nares into the stomach. The deposition of *Tubercle* in the lungs and bronchial glands is by no means an uncommon consequence of pertussis.

In reference to the influence the complications exert upon the progress of the disease, MM. R. & B. observe that this is very uncertain. When the complication is very grave the fits often diminish in intensity; but when this occurs only at a late period, it is often difficult to determine whether the disease has not become modified independently of its influence. M. Bouchut, however, following M. Trousseau, states that, intercurrent febrile affections exert a marked effect in diminishing the force of the disease, or curing it. "If they cause the patient to run some risk, by a happy compensation they moderate the symptoms of the principal disease." Among such affections he enumerates variola, acute catarrh, pneumonia, enteritis, meningitis, dental fever, vaccination, and *gourmes*. Complications are oftener observed in infants at the breast than in older children.

*Diagnosis.*—This, in the great majority of cases, is easy enough, except in the very early stages. Still, as MM. R. and B. observe, there is a paroxysmal form of bronchitis which may be mistaken for it, and we do not doubt its having been so, will explain many of the examples of reputed second attacks of pertussis. M. Bouchut places the diagnostic marks in the simplest manner by stating that in this bronchitis there is no whooping inspiration, no vomiting after the fit, and hardly any expectoration, while there is well-marked fever, which is rare in pertussis. A *tuberculous state of the bronchial glands* is also attended with a paroxysmal cough difficult to distinguish from hooping-cough. When pertussis is not readily cured, its third stage, or rather the dilated bronchi it has given rise to, may lead to symptoms hardly to be distinguished from *phthisis*, except by the result of the case.

*Nature.*—"While admitting that the nervous element plays a great part in pertussis, we cannot but believe that there is something in it beyond a mere lesion of innervation. A singular neurosis it is in fact, susceptible of transmission by contagion, prevailing epidemically, and attacking the same individual but once. The hooping-cough seems to us to at once partake of the characters of eruptive fevers and of those of convulsive diseases. Like the eruptive fevers it is contagious, epidemic, attacks children specially, is not subject to return, is preceded by a period of incubation, and by a precursory stage. Like convulsive diseases and simple neurosis, it is characterised by the marked intermission its principal symptoms undergo, by their special form, and by the absence of febrile action and anatomical alterations. It might be well termed a specific neurosis. It is a disease without an analogue, and if we wish to assign its place in the nosological list according to its affinities, it must be placed between the neuroses and the eruptive fevers."

*Treatment.*—There is sound sense in the following advice given by Rilliet and Barthez.

" Before enumerating the medicinal substances fitted to fulfil the indications the various stages of the disease furnish, we ought to advertize the practitioner that he will often find hooping-cough to be tedious, and resisting the best-devised plan of management. He must scarcely expect to find the disease cut short by his treatment, and the object he should have in view should be, not so much the abridgement of its total duration, as the diminution of its intensity and the prevention of its complications. Let the therapeutical agents be so managed that the most active shall not be those commenced with; replace certain medicines in a skilful manner by others, and in the employment of debilitant measures have a regard to the future—such are the councils whose utility can scarcely be too much insisted upon."

The early stage is to be treated as catarrh, not having recourse even to leeches unless positive inflammation arises. In the convulsive stage, *flour of sulphur* in doses from one to two grs. every two hours in milk, was recommended by Kopp, and has been found useful by Jadelot. Dr. Lombard speaks highly of the *carbonate of iron*, from 18 to 30 grs. being taken in the twenty-four hours. The *oxide of zinc* (in doses from 1 to 3 grs. according to the age of the child) has been found very useful by many; and, among others, M. Guersant speaks highly of it in very young subjects, increasing the dose to 15 or 20 grains. *Emetics*, given occasionally, have been recommended by all authors, and even very young children bear them well. Among the narcotics *belladonna* has been extravagantly praised by the Germans: but the French practitioners seem to have a much less high opinion of its deserts. M. Trousseau finds it most useful when combined with opium and valerian. M. Guersant attaches much importance to the use of *hemlock*, combining it with equal parts of belladonna and of oxyde of zinc, and giving $\frac{1}{5}$ gr. of the compound three times a day, to begin with. Both MM. Rilliet and Barthez and M. Bouchut discountenance the use of *revulsives*, such as blisters, stimulating embrocations, &c. When any important complication occurs the peculiar treatment of the hooping-cough must be suspended, and the new disease treated upon general principles.

Passing by the chapter upon *Spasm of the Glottis*, or *Laryngismus Stridulus*, which is scarcely recognised in France as a distinct disease, we may extract a few observations upon

### *Convulsions.*

" For a great number of years convulsions and worms enjoyed an entire predominance in the medicine of childhood: but in proportion as pathological rearches have extended, and the nosological table has been enlarged, so has the influence of these two morbid conditions been esteemed of less account. At present, the re-action has been even carried so far that many authors deny to convulsions the title of a disease, regarding them as a symptom common to many different affections. The same considerations which determine us to give a particular description of contraction of the extremities and essential paralysis, engage us to devote a chapter to convulsions. We shall see, in fact, those affections are symptomatic of a well-determined disease of the brain, sympathetic of a particular condition of the economy, or, lastly, they are primary, or at least in the present state of science, not referrible to any pathological cause. So with convulsions, we shall find them sometimes resulting from an appreciable cerebral disease, at others, developing themselves spontaneously, or in the course of affections of a very different nature, without our being able to establish any relation between the prior disease and the convulsion, and without pathological anatomy revealing any lesion of the encephalon appreciable to our senses. In a practical point of view we must insist upon the following divisions, which are capital. 1. Convulsions without lesion of the brain, primary, or sympathetic of another affection. 2. Convulsions symptomatic of lesions of the cerebro-spinal organ."

Of the 60 cases collected by MM. R. & B. in children, from 15 months to 11 years of age, in 35 instances the attacks were symptomatic (21 boys and 14 girls), and in 25 instances (15 boys, 10 girls), they were sympathetic. In the first, the convulsions marked the onset of the cerebral disease in 16 cases. In the other category they manifested themselves at the early stage only four times. Of 41 cases, occurring in children at the breast, observed by M. Bouchut, the convulsions were sympathetic or essential in 27, symptomatic of material cerebral lesion in 14. Of the 27 cases, 15 occurred during perfect health, and were all cured; and 12 occurred during the course of severe diseases, of whom 7 died, only one offering any cerebral lesion.

*Of the Diagnosis of these various causes* MM. R. and B. thus speak :—

" You are called to a child suddenly seized with convulsions. He is from one to six years of age, strong, robust, sanguine; the attack has followed a sudden fright, a blow, a fall, an indigestion; *i. e.* some appreciable occasional cause. What is the affection he is suffering from? You are in doubt whether the convulsion is primary, sympathetic, or symptomatic, or whether it is not a prelude to epilepsy. If the child was quite well, if the determining cause is well made out, if the constitution is good, and the fit not violent, you may suspect it is a primary or sympathetic convulsion, or an attack of epilepsy. You examine with care the various organs, and after you have assured yourself that there exists no symptom of pneumonia, pleurisy, &c. you hesitate only between deciding it to be a convulsive or epileptic attack, and are obliged to trust to the future for the solution of this doubt—acting in the mean time according to the urgency of the case, just as if you had to do with a case of simple convulsion. Suppose at the time of the attack the child was already suffering from severe disease, as pneumonia, pertussis, &c. In this case the convulsion is evidently sympathetic of the visceral lesion, is it at the same time symptomatic of brain disease? In the great majority of cases you can be certain it is not so. The brain suffers sympathetically, and not from any disease of its own substance which need cause you new alarm. But this is very different from saying that the convulsion is not dangerous.

" But it is quite different when the convulsion attacks a child the subject of a chronic disease. If you are informed that for weeks or months the child has been losing flesh, colour, and strength, has had a capricious appetite, irregular digestion, or vomitings; if you learn that its parents were phthisical, or that it has been brought up in hygienic conditions fitted for the generation of tubercular disease; then, although the convulsion may even be the result of an appreciable occasional cause, you must fear that the attack is but symptomatic of some grave cerebral affection, and deliver an unfavourable prognosis accordingly.

" If the child is more than ten years old the diagnosis is much easier; for it is very rare at this age for convulsions to be sympathetic or essential; and they are for the most part symptomatic of a disease of the brain, or constitute a first attack of epilepsy."

Upon the influence of *congestion* we have the following remarks :—

"Some authors have stated that eclampsia is always connected with a congestion of the brain or spinal-marrow. We repeat now, what we have already said concerning cerebral congestion, that it is often difficult to decide whether the hyperæmia has preceded or followed the attack, or whether it has coincided with it. In some cases we have found no trace of congestion, besides which, eclampsia may depend upon an anæmic state of the encephalon. What can we conclude from such contradictory facts but that congestion plays but a secondary part in convulsions.

"In this respect we partake of the opinion of the authors of the *Compendium*, who, after stating that, in most subjects who have died from convulsions, we find traces of congestion, inquire whether these lesions are cause or effect, and pronounce themselves in favour of the latter opinion. Nevertheless we do not mean to state that this is always the case. and we can easily conceive that a sudden congestion may produce a convulsive attack, just as we see this phenomenon result from an effusion of blood into the cavity of the arachnoid or the substance of the brain. But we maintain that frequently things come to pass in another manner, and besides the hyperæmia there is a lesion of innervation which is the proximate cause of the phenomenon. The solution of this problem is not a mere matter of curiosity. Practitioners have in fact but too great a tendency to treat all convulsions by bleeding—a practice sometimes useless and often fatal."

*Treatment.*—In accordance with the above principles, MM. R. & B. restrict bloodletting to the cases—1, When the convulsions are primary, the child robust, the fit violent, the face violet, the pulse small, or asphyxia or coma imminent. 2. When sympathetic convulsions, offering the same intensity, occur at the commencement of an inflammatory affection. 3. When the sympathetic convulsions are developed during the convalescence of an acute disease in subjects but little debilitated, or in the course of a neurosis. Where venesection is inconvenient, leeches, varying from two to fifteen, according to the age of the child, may be substituted, applying them to the temples or mastoid processes when much blood is required; or to the anus or malleoli, as recommended by M. Chauffard, when a derivative effect rather is sought. MM. Dezeimeris and Trousseau have particularly insisted upon the benefit to be derived from *compression of the carotid arteries*.

"M. Trousseau confines the employment of this means to cases of what he terms congestive convulsions, and in which the movements are perverted especially on one side only. He advises compression to be made in the interval which separates the sterno-cleidoieus from the sides of the larynx, for at this point the vessel is free, and it is easily flattened against the vertebral column. The compression is made with the thumb or index finger and the middle finger. The finger is placed parallel to the axis of the vessel, or perpendicularly, having the palm of the hand turned outwards so as not to compress the trachea or larynx. The compression of the carotids would prove hurtful rather than beneficial in convulsions occurring in anæmic children."

Derivatives to the extremities are highly useful, but in employing blisters or sinapisms we must never forget to watch their topical effects, since the loss of sensibility prevents any manifestation of suffering; and deep ulcerations, &c. have resulted from the neglect of this point. Purgatives, and, if the stomach is loaded, Emetics, are useful. The *oxide of zinc* has been highly spoken of as an antispasmodic, and M. Brachet unites henbane with it. A prolonged tepid bath is one of the best antispasmodics. *Narcotics*, as opium, must in children be most guardedly employed, and are only adapted to cases of long-continuance and attended with sleeplessness, or such as result from painful accidents, &c. *Tonics* are required in anæmic subjects. Free exposure to the fresh air, or placing the child quite naked upon a marble table, has frequently dissipated a convulsive attack.

### *Treatment of Chorea.*

MM. R. and B. after examining the claims of the four classes of remedies, antispasmodics, narcotics, purgatives and tonics, and insisting that, to be useful, each must be adapted to the indication of the particular case, and not administered, as is too frequently the case, promiscuously, present us with the following *résumé*, which we extract as an example of the recapitulation of heads of treatment with which they terminate each chapter of their work.

"A. A child seven years of age, habitually enjoying good health, muscular and sanguine, has been during a few days the subject of a chorea of moderate intensity. There is no complication, and all the functions are executed in a normal manner. Prescribe—1. A tisane of infusion of orange and lime leaves. 2. A sulphureous bath (this has in several cases been found rapidly curative) daily. 3. Light aliment, avoiding wine, coffee, and all excitants, and maintaining every hygienic precaution. If in about a fortnight there is no amendment, we may substitute an infusion of valerian for the orange tisane, and give, three times daily, a powder composed of four grains of oxide of zinc and 12 grains of sugar. If at the end of a month or more there is not amelioration, and the child becomes thin, we must employ the treatment next to be mentioned. B. A child who is weakly, with delicate skin and puny limbs, has been for several days the subject of an intense chorea. Prescribe.—1. A tisane of chenopodium, of hop, little centaury, of gentian, or of quassia. 2. Give from one to three of the following powders: ℞. Iron filings, gr. ij., Ext. opium $\frac{1}{6}$ to $\frac{1}{3}$ gr., Dry Ext. of Bark, gr. iv. 3. Three sulphureous baths per week. 4. Good diet. 5. If the case is tedious substitute carbonate of iron for the powders. C. A child of ten years old is attacked by an intense chorea, which allows him rest neither by night or day. The case has resisted the preceding treatment. Prescribe—1. The infusion of valerian as a tisane. 2. Three of the following pills, increasing their number according to their effects: ℞. Ext. Opium, Ext. Belladon. āā gr. iv.; Thridace, gr. 6; Powder of Mallows, q. s.; divide into 14 pills. 3. Give a purgative composed of calomel and jalap every four or five days. 4. A warm bath every third day. 5. Severe regimen."

We think that a smaller proportion of the simpler cases would prove rebellious if the authors made a larger use of purgatives, which they seem to reserve only for the obstinate ones. But the prejudices in France prevent this means ever having had a fair trial. On the other hand, we are certain the English practitioners do wrong in making no use whatever, in any affection, of any of the multitude of *tisanes*, used far too abundantly though they be by our neighbours.

*Contraction of the Extremities.*

Twenty-three cases of this affection form the basis of the authors' observations. It usually commences by a flexion of the fingers, which are also separated from each other and bent over the thumb. The wrists, and occasionally even the elbows, are also flexed; and when the affection is intense, the muscles acquire great rigidity, and the parts cannot be straightened without much force and pain. After a while the toes and feet become in like manner influenced. This affection is most likely to be mistaken for contraction, which is symptomatic of disease of the brain; but is chiefly distinguished from it by the rarity of accompanying cerebral symptoms (such as convulsion, strabismus, dilatation of pupils, &c.) preceding the contraction; by absence of irregularity of pulse; by being binary, and commencing in the fingers and toes instead of the knees and elbows; and by being remarkably intermittent. The prognosis is unfavorable, but this arises from the fact that the disease usually appears in the course of severe maladies, as pneumonia, bronchitis, enteritis, &c. which have produced debility, or this has been induced by onanism, or unfavorable hygienic circumstances. If the disease is treated by bleeding, revulsives, &c., it becomes much aggravated. Antispasmodics must be resorted to, in addition to the means proper for combatting the stage of the affection, during which it has become developed.

### Class VI.—Continued Fevers.

Under this term MM. R. & B. recognize febrile affections, which are not interrupted by intermissions, and which are anatomically characterized by a phlegmasia of the mucous membranes or of the skin, and whose point of departure is a general morbid condition, which probably results from some peculiar modification of the blood. Of this class of affections, however, the authors confine themselves to noticing *typhoid fever, small-pox, scarlatina and measles.* In typhoid fever the cutaneous eruption is not well marked as in the other forms of disease; but even in the eruptive fevers, the affection of the skin is far from always following a regular course, and in some cases does not appear at all. The affection of the mucous membrane is also more intense and more constant in typhoid than in the eruptive fevers—the cutaneous phlegmasia being the predominant symptom in the latter, the mucous phlegmasia in the former. Upon the *complications* we have the following remarks.

"These constitute in fact one of the most important portions of the history of continued fevers, and should especially engage our attention. They are numerous and should be placed under three heads. 1. Some depend upon the phlegmasia of the skin or mucous membrane itself, and the inflammatory element which doubtless belongs to these diseases. Such are all the acute inflammations which are induced, and certain dropsies which result from the perturbation of the functions of the skin and probably of the serous membranes. Thus we find bronchitis, pneumonia, anasarca, in fact the greater part of the affections which we have described in our first volume. 2. Those affections which we presume depend upon the intimate nature of fever, or rather of the lesion of the blood, as hæmorrhage, gangrene, and much more rarely, tuberculization. 3. Intercurrent diseases, which cannot be regarded as dependent upon the continued fever, but which, developed under other influences, complicate it and aggravate its prognosis. In this division certain phlegmasiæ, some dropsies, and especially the eruptive fevers themselves, are found. It is curious to observe how these diseases complicate each other, the same child simultaneously presenting a scarlatina and a rubeola or a variola. In this class of cases we must carefully distinguish the eruptive fevers which succeed each other from those which exist together. In the first case they may pursue their periods regularly; in the second, they become habitually irregular. But if the eruptive fevers complicate each other frequently, it is not so with regard to typhoid fever. It is extremely rare to see this disease developed at the end of an eruptive fever, which on its part never occurs during the febrile period of the *dothienteritis*.

"In the study of complications we can judge of the reciprocal influence of continued fevers and of the diseases which arise during their course. We shall find, in the following Chapters, that these secondary affections have their special marks; and it is not useless to seek what is the influence the eruptive fevers exercise upon the diseases during the course of which they appear. Now their effect is almost invariably that of aggravating those which appertain to the class of their habitual complications, while they may arrest the progress of such as do not belong to this. If, however, they do not cure them they in no-wise modify the evolution of the local lesion."

### *Typhoid Fever.*

This disease is represented by the authors as a very common affection in children; and many cases described by English authors as inflammatory affections of the abdominal canal with typhoid symptoms, and others described as remittent fever, are really examples of it. The most characteristic *lesions* are—(1), an inflamed state of the patches of *Peyer's* glands, which are softer, and much less disposed to pass into a state of ulceration, than in the adult; (2), an enlarged state of the mesenteric glands; and, (3), a tumefied state of the *spleen*, which, however, the authors represent as of far less frequent occurrence than M. Taupin states it to be. In 44 cases they found this organ considerably tumefied in 10, and slightly so in 8: but Taupin found it considerably enlarged in 109 out of 121 cases! Resolution of the inflamed patches has been frequently observed in examinations made at an advanced period of the disease. In only 15 out of 111 cases recorded were ulcerations found to exist.

We need not enumerate the *symptoms*, but may advert to what the authors say concerning the cutaneous *eruption*. This consists of small, round, flattened papulæ, disappearing on pressure, situated especially upon the abdomen, chest, and upper part of the thighs, and generally appearing between the 6th and 12th day, sometimes earlier, sometimes later. In most cases they are few in number, rarely amounting to 10 or 20 patches. They are not found in about one-third of the cases, and are sometimes seen in enteritis. The more severe the disease the less abundant are the spots in general, and *vice versâ*. Some time after the appearance of these spots, numerous *sudamina* manifest themselves, being especially found at the sides of the neck, but not usually continuing very long.

The authors admit the difficulties of the *diagnosis* of this disease from some cases of gastritis, enteritis, pneumonia, and meningitis, especially of enteritis. After detailing the distinctive marks at length, they observe—

"It results then from our comparison, that in certain cases it is impossible to distinguish typhoid fever from enteritis—a conclusion very different from that arrived at by M. Louis as regards the adult. In comparing the varieties of the one with those of the other, we are led to the conclusion that, in a considerable number of cases among the youngest children, these two diseases, about which there has been so much discussion, and which they have tried to separate, approach each other, and become confounded. These considerations are not idle, but should exercise an influence on our therapeutics. As soon as we are convinced that, in children from two to five years old, typhoid and enteritis resemble each other for the most part in their symptoms, we should avoid the employment of all means which, directed against the *dothienenterite,* may, by error, add a new stimulus to a disease simply inflammatory, and determine the eventual occurrence of grave accidents."

The distinction between typhoid and meningitis is also sometimes very difficult; and errors have been frequently committed by most distinguished pathologists and practitioners.

The authors arrange the various cases of the disease under three forms: slight typhoid; grave typhoid; and very grave typhoid. Of the 111 patients, there were 47 of the first, 41 of the second, and 23 of the last form; these three forms they also respectively term—(1), the mucous; (2), the bilious and inflammatory; and, (3), the adynamic and ataxo-adynamic. The *prognosis* of the first form is highly favorable, for only 1 out of the 47 died. The danger of the second form arises from the supervention of inflammatory complications, such as enteritis, pneumonia, &c. There were 15 deaths from among 41 patients. In the severest form, too, death is often hastened by a complication, although it may also result from the severity of the uncomplicated disease, which then kills through the excessive perturbation it has caused the nervous system, the intestinal lesions having frequently disappeared long prior to death. Of the 23 patients, 13 died. Thus, of the 111 cases taken together, 29 died. Pneumonia proved a very fatal complication, for of 22 children, in whom it supervened, but 4 were cured. In 9 cases anasarca was observed.

"We find that the *complications* of typhoid fever may become developed at very different periods of this pyrexia, but that in general they supervene at a but little advanced period. There exists in this respect great differences between the phlegmasiæ, hæmorrhages, dropsies, gangrenes, and the eruptive fevers. Thus, while the former of these affections may be developed during the period of increase, or of the state of *dothienenterite,* the eruptive fevers alone only appear during the convalescence, at an epoch when the febrile action has considerably diminished or entirely disappeared. It seems, then, that there exists, between the typhoid affection and the eruptive fevers, a repulsion the more remarkable inasmuch as these diseases resemble each other in so many of their characters. However, experience proves this repulsion is only temporary, as the eruptive fevers may appear the moment the typhoid disease has yielded the place to them."

Typhoid fever is especially observed between the ages of 9 and 14, less frequently between 5 and 8, and is rare in the earliest years of life. M. Taupin states that, in 121 cases he has observed the disease ten times in children of four years of age, but the cases given by all other authors range from 8 to 14. Of the 111 cases recorded, 80 were boys, and 31 girls; and of the 121 of M. Taupin, 86 were boys and 36 girls. Occasionally the disease occurs epidemically in the hospital; and M. Rilliet observed a mild epidemic in a village near Geneva, which exclusively attacked children, while, in a neighbouring village, numbers of adults fell victims to the same disease. The authors speak very doubtingly as to the *contagiousness* of the disease.

*Treatment.*—" Here, more than in other affections, we shall take care not to propose any special medicament—one that can be invariably applied always and everywhere, to slight and severe cases. As long as we do not know the intimate nature of typhoid, and that experience has discovered no antidote for it, we do not think it possible to lay down any treatment to the exclusion of all others. For this disease, as for the eruptive fevers, we are obliged to appreciate more or less particular details, which necessitate from day to day some modification in the plan."

It is to be borne in mind, that slight cases are almost always terminated by a restoration of health, and that it is to the complications we are to look as the source of danger in the severer forms; so that medicines having too debilitating an effect must be avoided.

The administration of *Purgatives* in typhoid fever has been much recommended of late years; but MM. R. and B. report unfavourably of their continuous use, since they, (1), do not influence the symptoms or course of the disease; (2), provoke inflammatory action in the intestine; (3), they do not prevent the development of complications, and indeed may favour it. They, however, do not object to an emetico-purgative at the commencement of the disease, and occasional aperients during its course. So, too, they object to *bleeding*, except in the case of robust children, in the early stage of the ataxic form. But, as a general means, it is improper, (1), as not influencing the symptoms; (2), aggravating them or generating others, and hastening the fatal termination; (3), favouring the occurrence of complications by the debility which is produced. It is more injurious in proportion as the disease has continued for a long space of time. The *Sulphate of Quinine*, which does not aggravate the local malady, is spoken of favorably in the adynamic forms. In most cases, hot vinegar or mustard *Cataplasms* were employed, and, in grave cases, *Blisters* were applied to the feet, although, according to our authors, with little effect on the cerebral symptoms. The *expectant* method, giving merely emollient and nitrous drinks, and applying cataplasms to the abdomen, and sinapisms to the feet, has proved of good service in several cases. The *Complications* require careful treatment, which need not be detailed.

### *Variola.*

The chapter of MM. Rilliet and Barthez' work on Variola is a long and very able one. We will cite a few of the most interesting particulars, first premising that the variolous eruptions are divided by the authors into five groups—normal or regular variola; anormal variola; normal and anormal varioloid, and varicella—this last being added in conformity with the observations of others rather than with their own. The anatomy of the variolous pustule is especially well described, but is given at too great length for extract.

" *State of Organs after Death.*—The lesions which have been found at the autopsy of subjects who have succumbed to variola are sometimes insufficient to explain death. In fact, we find some few kernels of pneumonia, a slight enteritis, a more or less entensive laryngitis, or even, lastly, an absence of all lesion of organs. But, after a more attentive examination, we find that there exists a more general fact than that just enounced, which is that, in most of the organs, and especially in the cavities of the heart and large vessels, a liquid, serous, blood, sometimes of the colour of wine-lees, in more or less abundant quantity, is found. If there are clots, these are often small, black, soft, diffluent; and it is rare to find them colourless and fibrinous. They are especially seen when an acute, intense, inflammation has invaded an important organ. In this case, sometimes fibrinous and solid coagula are found in the midst of an abundant serosity.

"If there are some exceptions to these remarks they are rare, and are almost always explained by the accessory circumstances. Thus, a patient in whom variola is complicated with gangrene, has presented at the autopsy a fluid and vinous blood, in spite of the co-existence of a severe and acute inflammation. The same is observed in children who have had several simultaneous or successive eruptions, in which case the presence of a pneumonia or pleurisy has not determined the formation of fibrinous coagula.

"To the preceding remarks we may add, that most of the organs present a more or less intense sanguineous *congestion;* the muscles are red and firm; the membranes of the brain much injected; the sinuses gorged with blood; the cerebral substance more or less injected. Abundance of blood flows from the pulmonary vessels when divided; the liver, spleen, and kidneys present the same aspect, the congestion being general. There are, however, children who offer an exception to this rule. We have seen several of these, in whom the eruption during life was pale, wan, and blanched like themselves, exhibit after death but a very moderate injection, or even a considerable paleness, of the various organs.

"The results of our remarks are then—1. The possible absence of all important organic lesion. 2. The sanguineous congestion of every organ. 3. The deterioration of the blood. This last lesion doubtless suffices to explain death; but in this view we may add to it, in a certain number of cases, the abundance of the eruption. So extensive a surface as the skin, and the mucous membrane of the pharyneo-laryngeal passages, cannot be inflamed over a large extent without danger to the patient."

The appearances which each organ manifests are then detailed, but we only extract those of—

"*The Intestines,* which present an aspect quite remarkable. Frequently there is considerable development of the follicles, either as regards number or size, at the commencement or end of the small intestine, and more rarely in the large. This follicular eruption consists in small hemispherical projections, either pointed or slightly flattened, and often presenting a small central black point, which is sometimes depressed. This may give rise to the belief of a vesicular or pustular development in the intestine, which seems to receive confirmation in the fact, that on pricking those projections, after carefully drying them, a little drop of serous fluid issues out.

"But this opinion is quite destroyed by the following considerations:—1. These same projections, containing serosity, exist in many other diseases, and are not special to variola. 2. A variolous vesicle cannot be formed where there is no epithelium to raise. 3. We have never seen the little false membranes, or other lesion, resembling those which are found on the other mucous membranes, as in the mouth, the pharynx, or larynx. The anal orifice is the only point of the intestinal mucous membrane upon which variolous pustules are found. 4. Lastly, another proof is found in the state of the patches of

Peyer's glands, which are as often developed as the isolated follicles. Numerous, large, projecting, softened, and often reddened, they exactly simulate certain typhoid patches at their commencement, and would be confounded with them if they presented any ulcerations. But we have never met with, and it is very rare to find, any enlargement, redness, or softening of the mesenteric glands. Nevertheless, this development of the patches and of the follicles is so frequent and so remarkable, that it ought to be taken into consideration; for it is one of the proofs of the tie which unites variola with typhoid.

"Besides this disposition, the intestinal mucous membrane frequently affords traces of an anterior congestion. Thus, it is covered with a layer of thick and adhesive mucus, or presents a general iron-grey colour, with punctations as if the blood in its passage had deposited its dark matter there."

*The Complications of Variola.*—These often add much to the gravity of the disease. Those discussed by the authors, at length, are—1. *Ptyalism,* which is rare in children. 2. *Articular Inflammations* and *Abscesses,* which have been observed only four times in 153 cases. 3. *Otitis,* another rare complication. 4. *Corneitis,* terminating in ulceration, of which four cases presented themselves, occurring at an advanced period of desquamation. 5. *Bronchitis* is a rare and unimportant complication. 6. *Pneumonia* is far seldomer met with in variola than in rubeola, and puts on a different form. The *lobar* is here much more common than lobular pneumonia. But *serous congestion* is the most marked characteristic of variolous pneumonia. "It is astonishing to see the quantity of sanguinolent serosity which the slightest pressure forces from the inflamed parts. This serous congestion often extends over the entire lung, even when there is little or no pneumonia—so as to form a true œdema of the lungs." It is a very serious complication when it occurs in the anormal form of variola; but is easily curable when occurring during the convalescence of the normal form. 7. *Intestinal lesions.* The dysenteric and chronic forms of these are more frequent in variola than in the other eruptive fevers, and the severe purging attendant upon them is a very grave symptom in children. 8. *Hæmorrhages* form perhaps the most dangerous of all the complications, and are by no means unfrequent; for they were found to exist in 12 out of 39 anomalous variolæ. Epistaxis, which is usually of the active form, occurring at early stages of the disease, must not be confounded with these dangerous passive fluxes. The eruption in these cases is always very anormal. Any organ may be the seat of the hæmorrhage, but it is rarely abundant without the skin participating. Next to the skin the various mucous membranes most frequently furnish the blood, then the lungs, and next the urinary apparatus and muscular system. From the third to the fifth day is the most common epoch of its appearance. When the skin is affected, the blood may occupy the pustules themselves, their circumference, or the intervals between them. The pustule must be opened to prove the existence of the small coagulum, and we must not confound this *purpura* with a mere violaceous injection of the skin. The prognosis in these cases is very unfavorable; and even a slight degree of this ecchymosis is a just cause of alarm, as exhibiting the serious change which has occurred in the blood. 9. *Gangrene* is a much less frequent complication in variola than in the other eruptive fevers. 10. *Anasarca.* Although œdema of the lung is pretty frequent in variola, it is not the case with the other forms of dropsy. Only three cases of anasarca are mentioned, two occurring at the end of the period of desquamation. 11. *Other eruptive Fevers*. The authors have never seen a normal variola existing at the same time with any of these; but they have seen cases of scarlatina or rubeola with anomalous variola and varioloid. One case is mentioned, in which rubeola and variola alternately suspended the progress of each other.

"Measles and scarlatina seem, at first sight, to determine a derangement in the regular course of the febrile movement of variola. Thus, if they are developed while this disease is in its stage of suppuration, the fever does not cease on the appearance of the variolous pustules, because the patient is under the influence of the fever which precedes the eruption of scarlatina or measles. If, on the contrary, the new eruption is only developed during the period of desquamation, the febrile action of the variola follows its normal course in the early stages; and a few days before the eruptions of measles or scarlatina, there is an augmentation, or a new development of fever, according to the state of the pulse during the variola. The disturbance of the febrile action is therefore rather apparent than real; for the fever which belongs to each eruption is developed at its proper epoch, that of measles, *e.g.* arising before or after that of variola, or coinciding with it, but not arresting its progress. The other symptoms offer a mixture of those which belong to each disease. Thus, bronchitis and lobular pneumonia predominate if measles becomes united to variola, while it is angina in the case of scarlatina. Here, there has been a double cause for the development of angina, and it has been constantly present in all our cases; but it has almost always been evident that the scarlatinous pharyngitis predominated over the variolous."

The complications which have been named are not found combined in the same individual, as in measles, in which three or four affections may co-exist. The intensity of the disease of the skin, and less susceptibility of the mucous membranes, may explain this.

"It will be seen that these variolous complications arise at two very different epochs; first, at the onset of the disease, not during the precursory symptoms, but from the first to the fourth day of the eruption, when the variola is anormal and mostly dangerous. Nevertheless, the anomalies are not always attributable to these complications, but to a predisposition existing prior to their development, and sometimes due to the epidemic constitution. The second epoch at which complications appear is that of convalescence, when desiccation is almost complete and desquamation has commenced, or sometimes at the end of this last. These affections, of a far less grave character than the preceding, belong, in general, to normal variola, and seem sometimes to act as a critical phenomenon —a complement of the eruption more favorable than hurtful. In other cases, however, this end is exceeded, the complication becoming really an unfavourable accident, having nothing critical about it, and is followed by death. In fact, among the normal variolæ, these complicated cases are those which prove fatal."

*Influence of Vaccination.*—The beneficial influence of vaccine in rendering any subsequent attack of small-pox milder, is well known; but the authors maintain it in some degree exerts the same effect even upon those who have never been vaccinated. For, although, as M. Bousquet observes, the varioloid disease has always co-existed with variola, yet the comparison of the accounts of variola in former times with what we see, assures us that the proportion of varioloid has very much increased. They think the generation of an hereditary influence by the operation of vaccination must be adduced to explain this; and the cases which came under their care 1837—40, were children of parents who had been born 1801—10, when vaccination was in full vogue in France.

MM. R. and B. do not recommend that vaccination should be at once practised when a child has been exposed to the source of variolous infection. It should be removed to another locality, until the time for the development of the variola, say a fortnight, has passed, when vaccination may be practised. This recommendation is founded upon the fact of their having observed, in several cases, not only that the course of the disease was not arrested by, but that a fatal form of variola occurred after, vaccination. This may have been a mere coincidence, for the children were young and feeble, and placed in adverse hygienic circumstances. One of the Sisters of the hospital, however, has remarked, that children vaccinated after remaining in the wards for some days, were generally brought back in a few days with a very severe form of variola.

*Prognosis.*—In reference to this, the authors do not attach the same degree of importance to the fact of the eruption being confluent or not that most observers do; but believe it of more consequence to observe whether there be complications or not. Simple variola is indeed usually curable, while the anomalous form almost always proves fatal. Of 39 examples only three were saved—this being the form in which change in the blood especially occurs. The younger the child the more unfavorable the prognosis, the anomalous and complicated forms being then most frequent.

*Treatment.*—The authors strongly protest against the system of generating the disease which prevails in the Paris Children's Hospital. Children are allowed to be brought into the wards where small-pox prevails, whether they have been vaccinated or not, and may remain there a day or two (or far longer) before this operation is performed, when they are again sent out probably to spread infection among their neighbours! Separate wards, at least, if not a separate institution, would seem an obvious precaution; but there can be no doubt that multitudes of children die at the Children's and Foundling Hospitals, in consequence of defective arrangements of this kind.

In regard to the *Management of the Eruption,* MM. R. and B. consider the point whether it is desirable to use any means to induce its abortion or favour its development, and conclude—1. That, in the great majority of cases, and especially when the eruption is normal, no general perturbating measures should be employed. The importance of having only a slight degree of eruption, stated by Sydenham, is less as regards the child, who succumbs generally to anomalous and complicated variola. 2. Nevertheless, Sydenham's observation, that variola is dangerous in proportion to the prevalence of the eruption on the face, is not to be lost sight of, and we may sometimes endeavour to lessen its force there by lessening the inflammation, and also thus preventing the disfiguring cicatrices. 3. Although nothing can be more judicious than Sydenham's prohibition of the heating and stimulating plan of treatment then in vogue, yet there are certain anomalous variolæ, in which the eruption is too slight and indolent; and then we must assist its development, as we may also when we wish to substitute an eruption on the extremities for one on the face.

As to the means of accomplishing the above ends, the authors do not approve of the administration of bleeding or emetico-cathartics in children, or of allowing them to rise from their beds while the fever is upon them, for the purpose of diminishing the amount of eruption, as recommended by Sydenham—this not being an object of the same consequence in the child as in the adult. In three instances, they tried the plan recommended by Eichorn, viz. vaccinating by 40 or 50 small incisions during the precursory stage, or at the earliest period of the appearance of the eruption. In two of these the eruption became irregular and the children died. In the third, both eruptions proceeded simultaneously, and far more rapidly than usual. The cauterization of the individual pustules by a pencil of the *nitrate of silver*, is an infallible means of causing their abortion when practised on the first or second day, although it frequently succeeds on the third, fourth, or even fifth day. "The inflammation as well as the pustule is cut short, at least this effect has never failed to follow our cauterizing the pustules at the edges of the eyelids. It seems incredible to behold how rapidly the œdema of these parts then disappears." It is, however, difficult to apply the caustic individually in the confluent disease, and in any case produces great pain, so that the authors agree with M. Rayer that its employment must be confined to those parts where it is important to prevent cicatrices, and on which the eruption is discrete. There is another plan whose employment is easier and nowise painful, viz. the direct application of the *emplastrum vigo cum mercurio* to the pustules. It should be applied the first or second day, or not later than the third. To be of avail a well-applied mask must be kept in complete contact with the face until the eruption has terminated upon other parts of the body. To meet the difficulty of maintaining the due application of the plaister in children, the apothecary of the Hospital has invented the following pommade as a substitute: ℞. Mercur. oint. 24 parts, Yellow wax 10 parts, Black pitch 6 parts. M. The application sometimes induces an eruption of *hydrargyria;* but is effectual in arresting the eruption and preventing scars.

We may observe that M. Goblin has published a paper in the "*Revue Medicale,*" June 1845, in which he states, that a recent epidemic at Stains has given him the opportunity of observing the great benefit derived from the application of mercurial ointment, as recommended by him in 1834. Treating the premonitory stage in a manner so as not to check the development of the eruption, when this has appeared, he applies mercurial ointment, using it stronger and more frequently in proportion as the eruption has a tendency to become confluent. This arrests the progress of the pustule, and removes surrounding inflammation—the ointment being continued for several days. No salivation or other general symptom occurs, but a rapid disappearance of the eruption on the face takes place, leaving no disfiguring marks. Two practitioners who witnessed this treatment certify to its success.*

The Chapter on *Scarlatina* does not contain anything calling for particular notice; but we may extract some interesting observations from that devoted to

### *Rubeola.*

*Complications of Measles.*—This is a very well-written section. Of all the complications that of *Broncho-pneumonia* is the most frequent. Thus in 167 cases of rubeola, MM. R. and B. observed 24 cases of bronchitis, several severe ones, 7 of pneumonia without bronchitis, and 58 of lobular broncho-pneumonia—several of the last, however, being slight. The bronchitis presents no special characters when occurring in measles, and dilatation of the bronchi has never been seen, unless the child had lived at least ten days after the occurrence of the inflammation. The pneumonia is almost always lobular, occupying both lungs to a greater or less extent —so that kernels of inflamed lung are found either isolated or united in every lobe—the posterior and inferior portions of the organ being those especially affected. Compared with pneumonia occurring under other circumstances, the formation of *abscesses* is very common in that of measles; nearly one-half the autopsies exhibiting these, and sometimes in large numbers. Broncho-pneumonia may arise at three periods of the disease,—1, during the precursory stage and the earliest days of the eruption; 2, during the decrease of the eruption; and 3, during convalescence. It occurs more frequently during the first of these than during the other two put together. When the pneumonia occurs thus early, it deranges the progress of the eruption, so that it is in the anomalous form of rubeola that it is generally found. The pneumonia which comes on during convalescence may be independent of the rubeola, and separated from it by an interval of good health, and is then often lobar: but in other cases it has a direct connection with the febrile disease, or follows other complications, and is then lobular. Sometimes the pneumonia becomes very chronic, giving rise to the supposition of the existence of tubercle, until the autopsy exhibits but abscess of the lungs. Broncho-pneumonia is one of the most frequent causes of a fatal issue of the disease; for, while in simple rubeola a return to health is the general rule, hardly one out of four or five survives this complication, which is, however, often combined with others of a fatal character. Its occurrence in secondary and anormal rubeola is much more dangerous than in the primary disease. Its production seems often due to a sudden chilling of the surface, accompanied perhaps with a retrocession of the eruption. Pneumonia is most common in the younger children, while bronchitis, which is seldom dangerous, is found in the older ones.

---

* Dr. Corrigan, in a recently-published lecture, speaks most highly, as a means of preventing future disfigurement, of the application of a compound, formed of lead plaister, melted with sufficient almond oil to allow of its being, when warmed, spread over the face by a pencil. It is allowed to dry, and the mask is retained until the scabs, detached by the new skin, gradually break it up and bring it away.

Among the other complications a frequent one is *Pharyngo-laryngitis.* In the 167 cases there were observed 24 of pharyngitis, 19 of laryngitis, and 16 of pharyngo-laryngitis. The authors believe this great predominance of these pharyngeal affections to be due to the simultaneous prevalence of scarlatina epidemics, as they regard laryngitis as a more special complication of measles than pharyngitis. There are the same lesions observed as these diseases produce in other cases, those of laryngitis being more serious, although merely the spasmodic form of this is also sometimes observed. This complication generally shows itself about the third or fourth day of the eruption, and does not generally add much to the mortality of the disease.

Lesions of the *Gastro-intestinal* mucous membrane are next in frequency to the pulmonic inflammations. Purging and swelling of the abdomen, however, must only cause the presumption of the existence of inflammatory action; for the autopsy has sometimes found, in cases where they have been present, the canal healthy. Nevertheless, the condition of the measles bears some relation to the extent of the diarrhœa. The course of the eruption is however seldom modified, although the febrile action is sometimes increased. This inflammatory affection is generally found in the anomalous forms of rubeola, and is accompanied by some other complication. It may show itself at the very commencement of the eruption, or some time after it has ceased, being generally more severe in the latter case than in the former. It is rarely the sole cause of a bad prognostic, but adds infinitely to the danger of a broncho-pneumonia. Injudicious food, or too active employment of purgatives, especially in young children, who are most liable to it, may induce this complication.

Another not infrequent complication is *Gangrene,* the mouth being the part usually affected, and after it, the lungs. It occurs from the thirteenth to the thirtieth day, and is rather an effect of the other complications than a direct consequence of the eruptive fever. Of eleven cases only one recovered.

Measles may likewise become complicated with *other eruptions;* and thus the authors have seen scarlatina seven times, variolous eruption twelve times, and erysipelas three times in conjunction with it. Such complications render the rubeola anormal, and its diagnosis becomes very difficult, the symptoms of the co-existing diseases becoming mixed. "A very remarkable thing in these cases is, that the intensity of the complications is the inverse of that of the eruptions. Thus, when the scarlatina predominates the bronchitis is most severe; but if the rubeola predominates the angina is most intense. This result, apparently so singular, is explained if we reflect that, in a great many cases, there exists a kind of balance between the phlegmasiæ of the skin and of the mucous membranes, so that if the one becomes diminished the other is increased; and thus the scarlatina, causing the disappearance of the rubeola, augments the gravity of the bronchitis."

The authors thus conclude their remarks on the complications:—

"Until now we have only endeavoured to appreciate the separate influence of each complication, and it remains to consider them when united. It is rare in fact for a case of rubeola to become complicated with but one affection; for in general several co-exist or succeed each other, so that death results only from an uninterrupted series of diseases. Broncho-pneumonia, pharyngo-laryngitis, and entero-colitis, are at once the most frequent complications of measles, and those which are most commonly found together. Broncho-pneumonia is as it were the centre around which the others are arranged. It is accompanied in fact almost as frequently by one as by the other, but the other two diseases are not often found together without a concomitant pneumonia.

"We have already stated the influence these complications have upon the progress of the rubeola, and that this becomes anomalous,—1, If a severe complication productive of febrile excitement is developed during the precursory stage or period of increase of rubeola. 2. If the rubeola appears during the course of another disease, or during the convalescence of a severe disease. * * * We have seen that complications occurring during the precursory stage prolong its duration and retard the appearance of the eruption: but when this shews itself, it will be irregular from its commencement, or will only become so one, two, or three days later, according to the epoch of the occurrence and increase of the pulmonary disease. Then, even, it will continue too pale, and will not take the coppery tint, or it will suddenly disappear between the second and third, or third and fourth day. The pulmonary inflammations, the most powerful of all in influencing the form of the eruption, are so, however, only when developed at its early stage, and before it has arrived at that stage in which it only disappears incompletely under pressure of the finger. It is seldom that the entero-colitis determines any variety in the course of the eruption. * * * * * * * * * Measles is considerably modified when it is developed during the course of another eruption, and that whether it comes on at the commencement of such disease, or after it is well established. In these cases its progress is more rapid, the period of decline becoming abridged, or altogether imperceptible, whether from its non-existence or its concealment by the other eruption. Lastly, the rubeola becomes anomalous when it appears during a severe disease which has deteriorated the constitution, after which, some time is required before the child can recover its habitual health, as in the convalescence of severe variola, scarlatina, or pneumonia. It is the same if the rubeola arises during the course of a slow disease which debilitates the child, as chronic enteritis, or tuberculization. In all these cases it participates in the blanching of the patient: pale, shrunken, and small in quantity, the eruption rapidly disappears. Sometimes it slightly re-appears in a few days, and constantly hastens the fatal termination."

*Influence of Measles upon Diseases in the course of which it appears.*— It generally hastens the progress of these or provokes a relapse, when the affection is among those which habitually complicate it. But if it is not so, the measles may arrest it, render it irregular, or even cure it. Thus it almost inevitably aggravates a pneumonia or tubercular disease; but under its influence a chorea, or anasarca after scarlatina, has been cured. In reference to its influence on *Tubercles* we have some useful remarks.

"This question is important, because its answer is contrary to the assertions we have made respecting the relations of tuberculization with the other eruptive fevers. Thus, we have seen that typhoid fever, variola, and scarlatina repel tuberculization, and we have sought our proof in the following facts—1. These affections do not engender tubercles. 2. They rarely attack tuberculous individuals, especially those in whom the cachexia has manifested itself. 3. They seem to exercise a favorable influence upon tubercle by causing it to pass into the cretaceous state. We believe rubeola obeys quite a contrary law, and have shewn that it is the origin of tuberculization in a considerable number of cases. We may add that it is not uncommon to see it developed in phthisical children.

We believe then that rubeola assists the development of new tubercles, accelerates the progress of those which are already deposited, and exerts no influence in causing them to pass into the cretaceous state."

M. Bouchut, speaking of younger infants, states the case as strongly.

"How many times have we seen, after this disease, pulmonary tubercles form in subjects who did not appear predisposed to them! How many times have we seen under its influence the tubercular affection, latent in the child, acquire a new force, and manifest itself with an unexpected rapidity! It is the fact that rubeola exercises a veritable power in the development of pulmonary tubercle, and much accelerates the progress of this affection in children who are its subjects."

*Prognosis.*—This is *favorable* when the disease is primary and the precursory symptoms do not continue longer than from two to four days. When the eruption commencing in the face gradually extends itself over the entire body: and the concomitant inflammation of the mucous membranes is not intense. When the febrile re-action is moderate. When the eruption increases during one or two days and then gradually declines, the fever, cough, &c. also diminishing and disappearing. When the child in losing its rubeolic aspect does not become excessively pale. When it asks for food, wishes to rise or play, or notices objects, &c. When the sleep is sound and natural. It is *unfavourable* when the precursory symptoms continue more than four days, and are accompanied with severe symptoms, as oppression, epistaxis, &c. When the eruption follows an anormal course, even when none of these anomalies have existed and the child has gone on well until the decline of the eruption, we must still fear complications and their consequences when the face continues red and flushed, or, on the other hand extremely pale; when the cough, dyspnœa, purging, &c. continue; if the nights are sleepless, and the child is dull, listless, and without appetite.

M. Bouchut quotes the following passage from that experienced observer M. Trousseau.

"It is a very prevalent prejudice among physicians, and especially in families, that rubeola and scarlatina are least dangerous when the eruption is most confluent. It is highly important to proclaim this a great and fatal error, for the influence of such a form of eruption is as hurtful as it is in small-pox. In this respect all our little patients (he is speaking of those in the epidemic presently alluded to) followed the general rule. All those in whom the eruption was discrete got well without difficulty. Three of those in whom it was confluent suffered from severe pneumonia; and what I observed in this little epidemic, affecting only one of my wards, I also observed in my private practice. It is especially however in scarlatina that this is evident; for while those cases in which the eruption shows itself chiefly in the throat and but little on the skin, are of slight gravity, those in which it manifests a bright colour and great tumefaction of the integuments, are as destructive as the most confluent small-pox."

*Incubation of Rubeola.*—The period of this has been very differently stated, by different authors. Of 38 patients who remained in the wards, the duration of the residence of whom prior to the appearance of measles was noted, in four instances the eruption appeared in from 4 to 5 days, in eight from 9 to 13 days, in twenty from 15 to 25 days, and in six from 28 to 58 days.

In reference to this point, M. Bouchut presents us with the history of a slight epidemic which occurred in the Necker Hospital in 1843, well observing that, more instruction has frequently been derived from the observation of epidemics in limited localities, than of those which attack large towns, where the history and progress of the various subjects are difficult of attainment. A child in the seventh day of the measles was brought into a ward in which were nine little children, one only of whom had had the disease. Of the remaining eight there were seven became affected, five of these 12 days after, and the two others on the 25th and 26th day. During the two months the disease prevailed, 17 other infants came into the ward, of whom two only took it, after remaining, the one 21 and the other 29 days, amid the infection. Succeeding new patients did not take the disease, nor did it pass into other wards, although these were only separated from this one by a boarded partition.

" It is then with the incubation of measles as with that of other contagious diseases, it has not the same duration in every subject. It would be rash to prescribe its limits, varying as it does in individuals and in conditions it is impossible to determine. Here the word predisposition offers a convenient disguise for our ignorance. In fact, according to predisposition, some are rapidly struck by an epidemic, and others are so only at a more distant period, or are even entirely spared by it. In other cases it is upon the form of the disease the predisposition seems to exert its influence. To recapitulate, in this epidemic the period of inoculation has varied from 12 to 29 days, a circumstance only explicable by the particular disposition of the children exposed to the infection."

This author quotes an observation of M. Guersant, which is of importance ; viz. that we must have regard not only to the natural but accidental predisposition. Thus the subjects of observation both in M. Bouchut's and in MM. Rilliet and Barthez' cases were already suffering from other diseases in their respective hospitals, which might exert a material influence in delaying the appearance of the measles.

### CLASS VII.—TUBERCULIZATIONS.

The all-important subject of Tubercle occupies no less than 600 pages of MM. Rilliet and Barthez' work, and is treated in a most masterly manner. The subject is here placed before us in first its general aspect, and then its particular phases, in a completeness hitherto unattempted ; and we only regret that our limited space compels us to curtail our notice more than we think desirable.

We think it best to transcribe some of the " Preliminary Observations" in the words employed by the authors.

" Tuberculization is the deposition in organs of an accidental production, without analogy in the economy, to which the name of Tubercle has been given. It is common to meet with this foreign body simultaneously in several organs, and whichever be that in which it is deposited, its nature, its evolution, and the greatest part of the phenomena it gives rise to, are the same. The different species of tuberculization, then, should, just as dropsies or phlegmasiæ, be considered as identical affections, whose differences principally result from the seat and functions of the organs they have invaded. Tubercle exerts on every tissue the same local action; thus very frequently occasioning the development of a secondary inflammation, of which the frequency and characters vary according to the nature of the organ affected. The tubercular phlegmasiæ form a group just as well marked as do the simple phlegmasiæ.

"The general pathological effects which tubercle causes, being only influenced in an indirect manner by the organs affected, present an almost perfect identity in all the species of tuberculization. The most common of these effects is a general wasting, a consumption, to which the name of phthisis has been given. Pathologists who were occupied specially with the diseases of adults had admitted several species of this; but the signification of the word, reduced from time to time, has at last been exclusively applied by M. Louis to pulmonary tuberculization. We believe that it is proper to reserve this word phthisis to express the consumption arising from tubercular disease; but we believe it is to restrict its use too much to apply it only to pulmonary tuberculization, while various or improper names are employed to indicate the consumption which follows the tubercular deposit in other organs. According to our view, the deposit of tubercles in an organ constitutes its *tuberculization;* the tubercular consumption which succeeds should be called *tubercular phthisis;* and the inflammation which precedes it, or is the consequence of it, *tubercular phlegmasia.* This important distinction exists already in part for some organs; thus we say tubercular pneumonia and pulmonary phthisis; but we shall make a much more extensive application of it, and speak of pleural tuberculization, pleural phthisis, and tubercular pleurisy, of peritoneal tuberculization, peritoneal phthisis, and tubercular peritonitis, &c. &c. The numerous relations which unite tubercular phlegmasiæ justify us in having passed them by in silence in our first volume. On the other hand, the easy generalization of tubercle, and the almost identity of all tuberculizations, lead to general considerations far more numerous than could have been presented in the former classes. Thus we shall give to our preliminary chapter a greater extension than we have hitherto done. By acting thus we shall have the advantage of not attaching to tuberculization of the lungs the general history of tubercle, a method too often followed, and especially faulty in regard to children. We shall, too, avoid repeating for each organ the same observations upon the pathology, &c. which are common to all; and we can offer the history of acute or chronic general tuberculization—a disease much more frequent in infancy than at any other epoch of life."

After employing various arguments to prove the *identity of scrofula and tuberculization,* the authors proceed to consider the subject of—

### 1. *Tuberculization in General.*

*The Pathological Anatomy* is elaborately given. The crude miliary tubercle or yellow infiltration is described as originating—1, in grey granulation (very frequent in the child); 2, gelatiniform infiltration, of which however but one example has been observed; 3, yellow granulations; and, 4, in what the authors term tubercular dust—so called from the exceedingly minute grains of which the deposit is made up, far smaller than that described as granulations. It may be disseminated amid grey granulation or a healthy structure.

The *semi-transparent grey granulation or infiltration* is regarded by many authors as the product of chronic inflammation; but the statement of Andral, that this is not found in any other organ than the lungs, is quite erroneous, as it may be seen in the child in every other organ of the body, liable to tubercle. MM. R. and B. believe that the grey infiltration may result from a chronic pneumonia, but that it may also appear independently of this. "In acknowledging the grey granulation may be an inflammatory product, we do not isolate it from the yellow tubercle, which has an intimate connexion with the grey structure. We may say that tubercular matter is yellow or grey, and that the latter may give rise to the former; and, in fact, it is only in subjects presenting the yellow tubercular matter that we observe the grey; the first may exist alone, but not the latter; and it is in the middle of this grey tissue that the yellow tubercle is habitually developed. The yellow tubercle is however at other times found without a trace of grey matter, and is then independent of inflammatory action."

We need not follow the description of the softening of tubercle, the production of cavities, and the transformation of their contents into cretaceous substance—this latter change being rarer even than in the adult. The natural tissue amidst which tubercle is deposited disappears, either from absorption in some cases, or from conversion in others. Even when it exerts no compressing or condensing effect, the tubercle will excite inflammation as any other foreign body, whence, indeed, in some measure, arises tubercular excavation. Such inflammation is not always eliminatory, but may be of a low chronic kind, favouring new eruptions of tubercles. At other times it is acute, inducing death much earlier than it would have resulted from the mere influence of the tubercles.

The tuberculous deposit varies somewhat in appearance according to the nature of the organ in which it occurs. Thus, *e. g.*, it has a more rounded form in the midst of parenchymatous structure, and is more flattened at the surface. Upon the serous membranes we find the small miliary form, and extensive patches or plates of tuberculous matter. The miliary tubercle and the yellow granulation are the forms observed in the mucous membranes, the grey texture being seldom there seen. The various forms of tubercle may be seen in the absorbent glands; but generally these consist of amorphous masses, which often attain an immense size.

The *distribution of tubercle in the different organs* is an interesting matter of observation, as shewing the differences which exist in children compared with adults, especially in young children. The various descriptions of tubercular matter are usually found in the same child, and thus, as in the adult, the lung, *e. g.*, may contain cavities, miliary tubercles, grey or yellow infiltration, &c.; but, in the child, a greater number of organs is usually simultaneously affected; and those organs, in which tuberculous matter is rarely deposited at a more advanced age, are far more liable to it in childhood.

"We believe we may divide our patients into three categories. In the first, the partial or general tuberculization is very abundant, and has been the principal or only cause of death. These constitute a little more than half of the cases. In the second, we place the children who had but a small number of tubercles, which cannot have contributed to the fatal termination—these forming more than one-fourth. The others, not constituting a fourth, form an intermediate class, the tuberculization in these being of a medium intensity, and having contributed, though but in a slight degree, to the fatal termination. Nevertheless, in nearly half of these cases, the tuberculization, although slight in any particular organ, has invaded a considerable number, and enough to entitle it to the term general. In 312 autopsies, 162 were found to have a considerable partial or general tuberculization, 64 a tuberculization of medium intensity which was general 26 times. In 86 instances there was a deposit of a small number of tubercles in one or in several organs. In examining these three classes in reference to the age of the children, we find the slight tuberculizations are chiefly found in those aged from 3 to 5½; the medium tuberculizations from 1 to 2, and then from 3 to 5: while the very abundant are found oftener from 6 to 15 than from 1 to 5 years of age.

"In the child, as at a more advanced age, the lung is the organ which of all others oftenest tuberculizes; the bronchial glands come next, then, but at a great distance, the mesenteric glands and the small intestine. After these organs, the pleura and spleen are oftenest invaded, and then the peritoneum, liver, large intestine, membranes of the brain, the kidneys, the brain, the stomach, and the pericardium. Nearly the same order prevails as to intensity as to frequency; that is, the organs which are oftenest tuberculized, are those also in which tubercle is deposited with most abundance. The principal difference is found in the mesenteric gland and small intestine. Abundant tuberculization is much more rare in the former, although its existence is somewhat more frequent than in the latter. * * * * The tendency there is in several of the organs to become tuberculized at the same time is great; and if it is true that there are a good many cases in which only two or three organs contain tubercles, it is also true that, of the fourteen organs we have specially studied, we have frequently found from four to eight so affected, and have even seen as many as from ten to thirteen. There is scarcely but the lungs and bronchial glands which form an exception to the rule. It is rare for them both to be tuberculated at the same time that the digestive tube or mesenteric glands are so alone. It is not rare to see these four organs simultaneously tuberculated, to the exclusion of all others. M. Louis has stated that, in the adult, we never observe tubercles in any viscus, without finding them also in the lungs. This law is not so true in infancy, for of the 312 cases, the lungs were found to be, after a minute examination, exempt from tubercle in 47 instances; and M. Papavoine has shewn the lungs were exempt from accidental productions in 12 out of 50 tuberculous children. * * * * * We may terminate these observations by stating, that it is common enough to see phthisis exclusively thoracic, and not rare to see it exclusively abdominal. Lastly, it is sometimes only encephalic, and it is the exception to find an advanced tuberculization at the same time in the abdomen and head, to the exclusion of the chest."

The above and other deductions are illustrated by detailed tables.

*Symptoms.*—The *face* is usually remarkably pale, although sometimes partially flushed, or rendered violaceous, if the respiration is obstructed. *Fever* is almost always present; but the pulse may rise to 140 or 160, without much heat of skin. Intense or not, according as the case is acute or chronic, the fever once established persists, and becomes much aggravated upon the supervention of any inflammation. The *skin* is dry and harsh, and partially desquamates, the patient being nevertheless much tormented with severe *sweating*, either partial or general, which seems to bear no relation to the presence or absence of diarrhœa. Partial *anasarca*, in a greater or less degree, is very common, being chiefly induced by the pressure exerted upon the vessels by the tubercles. *Wasting* is one of the most constant symptoms, and is not unfrequently the only early one of the disease. "Whenever a child more than five years of age wastes away, without this having been caused by an acute disease, for a considerable time, we must always suspect tubercle." The derangement of the digestive organs will be best considered in abdominal phthisis, but it may be mentioned that the *tongue* is usually normal but very pale, except when there is inflammatory complication, when it may become red, dry, &c. The examination of the *abdomen*, when the organs it contains are suffering from no lesion, produces but negative results—an important fact, since, where the abdomen is found to have been distended and painful for a certain period, we may certainly conclude that lesions do exist. The functions of the organs may however be considerably deranged, independently of any lesion detectable after death, as in intense and prolonged diarrhœa. The desire for food and drink is more influenced in acute than in chronic lesions, and in those of the lungs than of other organs. But some children, even where there are lesions of the intestinal tube, maintain a natural appetite to the last.

Tuberculization may be acute or chronic as regards its duration or the severity of the symptoms. The *acute* form is generally characterized after death by grey or yellow granulations, or small isolated miliary tubercles, existing in a greater or less number in all the organs. They are rarely softened or cretaceous. Although one organ may be more especially affected than another, the tuberculization is usually general. It may arise spontaneously, or under the influence of an acute disease, especially measles, and is frequently accompanied by acute pneumonia. Whether primary or secondary, it may manifest itself under two distinct forms, the simply febrile and the typhoid. Its duration is comprised within rather vague limits, viz. from 16 or 18 to 60 or 80 days. Meningeal tuberculization usually passes through its course with most, and pulmonary tuberculization with least, rapidity. The *chronic* form is characterized by the presence of all the species of tubercle in their different stages. Hence the local symptoms are much more distinct. Its duration is very unlimited, generally, however, occupying from three to seven months in passing through its course.

*Causes.*—An infinity of these have been assigned for the production of tuberculization, but the authors only notice such of them whose influence they have been in a condition to examine. To this end they have noted the autopsies of 525 children dying in the hospital, in 314 of whom tubercles were, and in 211 were not, detected. First, in regard to *hereditary transmission*, this has been admitted by all authors; but the authors believe that Lugol carries the doctrine much too far, confounding congenital predisposition with direct hereditariness. The figures collected to elucidate this point are few. Of the 525 children, the probability or certainty of their parents having been tuberculous, was ascertained only in 62 instances: that they certainly or probably were not so, appeared in 233 instances; while no information could be obtained in 230 cases. Of the 62 instances, 16 were children who died non-tuberculous. Of the remaining 46 children, who died tuberculous, the disease seems to have been transmitted rather more frequently by the father than by the mother.

2. Among the *anti-hygienic causes*, a frequent cause is found to be the vitiation of the air; but although many of the children who died tuberculous had been exposed to the privation of air and light incident upon miserable domiciles, so had many of those who died non-tuberculous. M. Baudelocque has exaggerated the influence of this cause; for "all those who have lived in a vitiated air do not become scrofulous, and all the scrofulous have not lived in any such air." It is a powerful cause, but not an indispensible one, and requires the co-operation of other causes. The authors believe that *humidity of the air* facilitates the contracting tubercle; and attach more influence to *bad nourishment* than Baudelocque has done, although the tuberculous children subjected to it, examined by them, only slightly preponderated over the non-tuberculous. *Onanism* is a habit frequently found in the children brought to the hospital, especially the tuberculous. It is an error to suppose that any one of these or other anti-hygienic causes will alone induce scrofula or tubercle: but when one or more are united, their influence is undeniable.

3. *Influence of Prior Diseases.*—(1) *Phlegmasiæ.* It is still a subject of dispute with the most eminent pathologists, whether inflammation can give rise to tubercle. The authors reply unhesitatingly in the affirmative; but add that, although it may be the immediate cause of this, it will only produce the deposition in those already predisposed; for "a pneumonia no more creates tubercles by its sole influence, than a chill creates a pneumonia or articular rheumatism." The tubercle may be deposited in the locality of the inflammation or in a remote organ, the progress of the affection being chronic in the latter case. Pneumonia and entero-colitis are the phlegmasiæ which especially determine the deposit, chiefly, however, in persons debilitated from other causes. (2) *Pertussis* without doubt may, aided by other causes, give rise to tubercle, which is usually first deposited in the bronchial glands. (3) *Typhoid Fever* and tubercle seem for the most part to possess a mutual power of repulsion. (4) *Variola* seems to possess little power in inducing or preventing tubercle. In none of the authors' variolous patients did tubercle supervene, nor did any of the chronic cases of the 314 tuberculous patients succeed to small-pox. Children who have had the small-pox are, however, by no means exempted from tubercle. (5) *Vaccinia.* The authors state, as the result of their investigations, that *vaccinated children are more disposed to become tubercular than unvaccinated children.* "We do not, however, state that vaccine is a cause of tubercle; but only that vaccinated children die oftener tuberculous than non-tuberculous, and that the contrary is the case with unvaccinated children. We conclude, therefore, that it favours the predisposition." (6) *Scarlatina.* This, like small-pox, exerts little effect in favoring or preventing the development of tubercle, but like it, it impedes its march when already formed. (7) *Measles* is a direct cause of tuberculization; and even children who have long since become completely cured of this disease are more liable to tubercle than those who have never had it. (8) *Chronic Disease,* such as tedious intermittent fevers, disease of the heart, rickets, &c. may give rise to tubercle. The tubercles, however, in these cases, are never in great quantity; and the number of non-tuberculous children dying from the same influences, exceeds that of the tuberculous. Rickets, indeed, has not been found a cause of tubercle, although several ricketty children have presented the accidental product. The coincidence of the disease is by no means so rare as represented, for the authors have found it three times out of seven in ricketty children.

"But, as we have often said, it is common to see these various causes uniting in their operation upon the same child. Thus, if a pneumonia or rubeola produces tuberculization it is in a child whose parents have died tuberculous, or who has been long exposed to anti-hygienic causes. It is by losing sight of this fact that so many pathologists have believed in the exclusive influence of certain morbid agents. The anti-hygienic causes are certainly those which are, when existing alone, the most powerful: but then several of them act simultaneously. On the contrary, hereditariness and the various acute and chronic affections more rarely suffice for the production of the disease."

4. *Age & Sex.*—M. Papavoine, from numerous data, has concluded that tubercles are rare from birth to three years of age; increase in frequency from 4 to 7, and about the epoch of puberty they are about as frequent as from 3 to 4. He has erred, however, in reckoning the age of the children from the period of their death rather than from the onset of the disease. According to MM. R. and B. tubercle is especially frequent from 6 to 10½; then from 11 to 15; then from 3 to 5; and lastly, from 1 to 2½. This calculation is made comparing tuberculous with non-tuberculous children; but if we confine ourselves to the former, they are as numerous from 3 to 5½ as from 6 to 6½; and nearly as much so from 1 to 2½ as from 11 to 15. As to *sex*: of the total, 314 tuberculous children 195 were boys, 119 girls. From 1 to 2½ the boys are far more liable to the disease, while the difference is with the girls to some extent from 3 to 5. From 6 to 10½ the sexes are equal; but from 11 to 15 the girls are more liable.

5. *Conclusion.*—"We may group as exclusively predisposing causes, a weak constitution, the age from 6 to 15; the feminine sex and vaccination. Those which act either as predisposing or as occasional causes are hereditariness, vitiation of the air, residence in a damp locality, bad nourishment, prolonged residence in a hospital, onanism, some of the phlegmasiæ, pertussis and measles. In enumerating so few causes compared to the great number we alluded to, we have confined ourselves to those whose influence we had it in our power of demonstrating in children from 1 to 15 years of age. We may remark, in fine, that most of these causes are debilitating; and that the others, at first exciting, are followed by the formation of tubercles, in consequence of the secondary debility they induce."

*Treatment.*—As tuberculization when confirmed is difficult, if not impossible, of cure in the great majority of cases, every effort should be made to prevent its development where its occurrence may be suspected; and, in this point of view, a knowledge of the causes is of great importance. For the *Prophylaxis,* then, we have to place the patient under the best hygienic circumstances we can command for him, and which are detailed at some length by the authors. The treatment of the acute diseases which may give rise to tubercle, should be (1), active but not too long continued, so as to avoid a tedious and troublesome convalescence. (2) It should not be too debilitating. (3) Tonic treatment should be promptly put into force. Masturbation is often prevented only with the greatest difficulty. It occurs even in very young children, and is favoured by warm, soft beds. It is sometimes induced by herpetic and other affections of the prepuce and labium, or by the irritation propagated by worms at the extremity of the rectum. Donné has shewn that the urine of these children, examined microscopically, shortly after indulgence in the practice, contains mucous matter mixed with crystals of oxalate of lime. Of the *Curative* treatment there is not much to be said. The authors enter into an examination of the various medicinal substances, as iodine, &c. but without adding any original observations upon their effects.

2. *Tuberculization of the Bronchial Glands* (*Bronchial Phthisis*). This disease, special to childhood, is of frequent occurrence, and is important, both on account of the severe consequences it gives rise to, and the obscurity it sometimes imparts to the diagnosis of pulmonary tubercle, with which it is very frequently associated. All the varieties of tuberculous matter may exist in these glands, but that of infiltration is by far the most common. The number of glands affected varies much in different subjects, from five or six surrounding the bronchi, to a vast quantity—the more deep-seated never acquiring so large a size as the more superficial ones. The enlarged glands, by compressing the vessels, may give rise to hæmorrhages or œdemas, or, more frequently, may narrow the calibre of the trachea or bronchi. Perforation of the bronchi when, and sometimes before, the tubercle softens may occur; and doubtless the cysts formed by these intercommunications have frequently been mistaken for true vomicæ. In 249 autopsies, perforation has been observed in 27 instances. Perforation of the blood-vessels or œsophagus is very rare.

No author has noticed the interesting fact, that tubercles occupying the lungs and those occupying these glands have a tendency in their progress to approach each other. There is a great disposition to the deposition of tuberculous matter upon the surface of the lung, which, however, after a while, instead of augmenting in quantity there, projects towards the parenchyma of the organ, at the same time that the glandular tubercle is pursuing a similar direction—so that they soon effect a junction. This occurs when the tubercles are yet crude; and, in the same way, when they become softened, the respective cavities tend to unite.

*Symptoms.*—The symptoms distinctive of bronchial phthisis are ascertained with some difficulty, since it is very rare for this form of tuberculization to exist alone. The compression produced on the vena cava by the glands, when very large, may give rise to œdema of the face, dilatation of the veins of the neck, or a violet-coloured complexion. When the pulmonary vessels are so compressed we may have hæmoptysis, which is however very rare in children. Pressure of the pneumogastric nerve also may give rise to a *spasmodic cough*, and asthmatic paroxysms: and the voice may undergo much change, being at first hoarse, and then muffled or extinct. By auscultation, a very large, loud, sonorous rhonchus is heard, of remarkable persistence; and if humid rales exist, as in a bronchitis, these may become augmented so as to become more or less a gargouillement. There is also great weakness of the respiratory murmur, especially at the upper part of the chest. We may, indeed, hear all the sounds audible in phthisis, from prolonged expectoration to cavernous breathing, even when there is no communication with the bronchi. It is chiefly by the different epochs at which they appear, and their irregular character, that these sounds are distinguished from those denoting phthisis. When the lung is itself tuberculous, the affection of the bronchial glands much modifies the auscultatory signs produced by it—exaggerating the anormal sounds to a great extent. So, too, the lesions of one lung may be the cause of the transmission of sounds to the other, giving rise to the fear that they are both affected. Dulness of percussion may likewise also lead to error, unless compared carefully with the auscultatory signs. The authors observe that, before they were aware of the above facts, they committed numerous and striking errors of diagnosis in these cases. "Then we found that the ideas, or rather the words, adopted since the time of Laennec, were not entirely applicable to children. Thus the *cavernous respiration* exists in the child in other lesions as well as when cavities are present, while under certain conditions and influences it is absent when they are present." The authors believe that the greater propinquity of the ear to the parts examined in the child explains this augmentation of sounds.

"The various symptoms which we have enumerated, and which are the result of the action of the enlarged and hardened glands upon the vessels, nerves, bronchi, and lungs, do neither exist constantly or all united. Their existence depends upon the position of the glands and upon their development. But even when they exist they are subject to a remarkable law of intermission, from which none escape. Thus, œdema of the face appears and disappears easily. The changes of the sound of the voice and cough, the paroxysms of the latter, and the fits of asthma, exist one day and disappear the next, to re-appear at some indeterminate epoch. The stethoscopic signs are not constantly the same, and do not observe a regularly increasing progress. Thus one day we perceive evident bronchial respiration, the next day only prolonged expiration, and the day after, cavernous respiration ; so that dull respiration, prolonged expiration, bronchial and cavernous respiration, pectoriloquy, gargouillement, and even sonorous *râles*, may alternate or succeed each other without any regularity at uncertain periods."

In the great majority of cases bronchial phthisis pursues a very chronic course. The *diagnosis* must be derived from an attentive consideration of the above symptoms, and is often difficult. Besides the intermittence of the physical signs above mentioned, the locality in which they are heard must be taken into account.

"The signs of bronchial phthisis are almost exclusivaly observed at the upper part of the lung, and principally on a level with the root of the bronchi, in the interscapular space. They exist also sometimes in front, but far more rarely. In the adult such a distinction as this would prove of little use, as tubercles almost exclusively occupy the apex. It is not so in the child, for they are often disseminated, and when they are seated at the apex, it is especially under the clavicle they are detected by auscultation. When then in a child suffering from a chronic pulmonary affection, we find signs of tubercle in the inter-scapular spaces, we should be led to believe, if these are variable in their progress and intensity, that they depend upon tuberculization of the bronchial glands."

In reference to the *Causes*, there is little special to be added to what has been said in respect to general tuberculization. Measles and pertussis especially induce glandular or pulmono-glandular phthisis. In all probability, also, chronic inflammation of the glands may give rise to the deposition of tubercle. Probably, too, inflammation of the lungs or bronchi may induce inflammation and tuberculization of these glands ; but upon this head nothing very positive is known. All the periods of infancy are nearly alike liable to this form of tuberculization, the youngest being perhaps a little more so than others. Very advanced tuberculization is however chiefly observed between the ages of 6 and 15. Girls are less subject than boys before 11, but from 11 to 15 the disease is as common in one sex as the other—the very advanced form being however at this age more common in girls.

### 3. *Tuberculization of the Lungs and Secondary Lesions (Pulmonary Phthisis.)*

The pathological anatomy is detailed at considerable length, but we must content ourselves with a summary of results. There were 265 autopsies. In 94 of these, grey granulations, and in 42 grey infiltration were found—the latter becoming much more proportionally frequent as the child was advanced in years. Chronic pneumonia, which is only found in tuberculous children, and which the authors consider as the transition between acute pneumonia and tubercle, existed in 19 instances. The tubercular dust occurred 15 times; but only twice as the sole tubercular deposit. In 68 instances, yellow granulations were met with: but the miliary tubercle is by far the most frequent form, 164 presenting it—more than a third of whom had but this species of tubercle. In 88 instances, the yellow infiltration existed. In 39 cases, softened tubercle was found, occurring especially between the ages of 11 and 15. In 77 cases, cavities existed. In 21 instances, cretaceous conversion of tubercle had taken place; and in 8 cases, cicatrices were observed. We may extract the following general observations.

"The lungs are very often tuberculous in the child, but less often so than in the adult. Thus, of the 312 children who form the subjects of our observations, 265 had tubercles, and 47 were entirely without them. It is especially from the age of 3 to 5½ that the lungs of children are found healthy. We find in fact they are so 1 in 4 or 5 times; while from 1 to 2½ they are healthy but once in 8; from 6 to 10½ once in 10; and from 11 to 15 once in 7 or 8. Tubercles are a little more frequent in the right than in the left lung. This slight difference continues to the 6th year, while after that age there is a slight predominance in favour of the left lung. In much more than half the cases both lungs were affected: but the younger the child the less frequently did this occur. Lastly, let us recal an observation we made in our first volume, that of the whole number of children whose lungs contain tubercle, the tuberculization in more than half the cases is a secondary affection. This applies especially to the younger children, and is much more rare in those aged from 11 to 15. Among other children, again, the pulmonary tuberculization is very abundant, and is evidently the principal cause of death. This is so with more than a fourth of the cases; the proportion being considerable between the years 6 and 15, and especially so between 10 and 15. In a third category may be placed the children in whom the pulmonary disease, though intense, would not have sufficed to cause so speedy a death. This comprises less than a fourth of the cases, and no considerable difference as to age being remarkable."

Among the *Secondary Lesions* may be especially mentioned a considerable *œdema pulmonum*, *bronchitis*, and *acute pneumonia*. This last lesion is the most common by far. It may be caused by the direct irritation produced by the tubercle, and is then usually lobular, or it may arise in a distant part of a tubercular lung, when it is usually lobar. Lobular pneumonia is most common prior to 10 years of age, attacking the right lung especially and frequently both. Of 69 cases in 52 instances both lungs were affected, in 11 the right, and in 6 the left alone. "In comparing the two diseases we find there is a great number of children who have both pneumonia and tubercle in one or both lungs; a less number who have tubercles without pneumonia; and a yet less number, pneumonia without tubercle." Of the 312 children in 11 only were the lungs found to be devoid of one or other disease.

The *Physical Symptoms* of the disease are detailed in a masterly manner, but at such length as to defy analysis in the space we have left. Of the *Rational Symptoms* we may mention that the *Respiration* is rarely natural in a phthisical child; the inspirations reaching sooner or later to from 30 to 80 per minute; being sometimes performed with ease, and at others with great labour and anxiety. It is rare to find acceleration at the commencement, unless there is a pneumonia also; and it is never so considerable in chronic as in acute tuberculization. *Cough* is nearly a constant symptom. It is often the first indication of tuberculization, which may have affected other organs, having invaded the lung. The cough may put on various characters, but usually continues during the whole period of the disease. When it is dry, small, frequent, or continuous, it indicates disseminated crude tubercle, and it becomes moist as the disease proceeds. When the cough is paroxysmal it must be distinguished from pertussis, which it is by the absence of whooping, less congestion of the face, the less suddenness of the attack, and the absence of viscous discharge upon vomiting. Such paroxysms occur when the bronchial glands are tuberculized or when mucosities become accumulated in dilated bronchi, or in cavities; the former being their exclusive cause in acute phthisis. *Expectoration.* Very young children do not furnish this, but those from seven to fifteen years do so not unfrequently, but never unless there is a cavity, pneumonia, or bronchitis. In five children only did *hæmoptysis* occur, and in none of these at an earlier age than seven. The symptoms in the child do not pursue the same regular course as in the adult. "The distinction between those of the commencement and those of the second period is difficult to establish, and frequently cannot be determined precisely. Nor can the symptoms always aid us in distinguishing the second from the third period. We know, in fact, that great enlargement of the glands suffices to lead to the belief of the existence of cavities." Although the disease generally runs its course in between three and seven months, it may occupy more than a couple of years in doing so.

In treating of the *causes* of pulmonary phthisis, MM. R. and B. offer a few additional observations upon the influence exerted by inflammation. They maintain that, although this is not a necessary preliminary, since tubercle is deposited independently of it, yet that in other cases the relation of cause and effect is undoubted—the predisposition being admitted to exist. M. Louis's observation, that pneumonia is developed at the base of the lung, and is simple; while tubercle is found at the apex, and is generally double, loses much of its force in the young; for in them tubercle often attacks the base and pneumonia is often double. But, granting the base to be more frequently inflamed, it is not necessary to admit that pneumonia and tubercle follow the same laws, to believe in the relation of cause and effect; and all we can say is, that when pneumonia occupies the base it has less tendency to terminate in tubercle. "The existence of such predispositions explain the rarity of the fact. In order that a pneumonia may terminate in tubercular deposit, it is required (1) that it shall be seated at the apex; (2) that it occur in an individual predisposed to tubercle; (3) all the individuals predisposed to tubercle do not acquire a pneumonia of the apex. These conditions render the fact rare, but it is no less real. Pneumonia is here but an occasional cause of an imminent disease; as a proof of which, tubercle once deposited in an inflamed part, soon extends itself and becomes general."

Pulmonary phthisis occurs with nearly equal frequency at all ages of childhood. But advanced tubercle is more commonly found in the older, and crude tubercle in the younger, children. The disease is also more frequent in very young boys than girls, but the contrary about the epoch of puberty.

### 4. *Tuberculization of the Pleura* (*Pleural Phthisis*).

There were 109 children who presented this form of the disease, and the following is the authors' summary of their detailed account of the *pathological anatomy*.

"The tubercular deposits may take place upon the external or internal surface of this membrane and give rise to the following forms:—1. In the great pleural cavity the tubercles may form large patches which compress the lungs. 2. Externally to the membrane are produced these tubercular patches, or more or less considerable cavities. 3. The intra-pleural tubercles very rarely soften, and we have never seen them perforate the pleura. The extra-pleural may soften and ulcerate the serous membrane. 4. They may form free communications with the bronchi. 5. When these are in contact with a tubercular infiltration at the surface of the lung, there is first produced a pleuro-pulmonary mass of tubercle, and then a cavity, which commences at the same time in the lung and the costal pleura. 6. If the pulmonary tubercles were already united to the bronchial glands, a mass of tuberculous matter traverses the lungs from these latter to the external surface of the costal pleura. 7. The different species of tubercle are, as regards frequency, as follows; miliary tubercle and tubercular patches; yellow granulation (which has been mistaken for false membranes), and grey granulation. 8. Tubercle is commonly deposited in both pleuræ, and when but in one that is frequently the right. 9. Considerable tuberculization is usually unilateral. 10. Intra-pleural is more frequent than extra-pleural tubercle, and it is rare to find the two conjoined."

*Pneumo-thorax* is not very rare in the child, since seven cases were observed by the authors. Perforation of the vomicæ are, however, much rarer than in the adult. The pathology, symptoms, &c. are described at some length.

### 5. *Tuberculization of the Pericardium.*

Tuberculization of the pericardium has been only observed 10 times in the 312 autopsies, and in two of these was not much advanced.

### 6. *Tuberculization and Ulceration of the Larynx (Laryngeal Phthisis).*

It is very rare to find tubercles deposited under the mucous membrane of the larynx or trachea; and the ulcers, which are rather common, differ from the ordinary ulceration of the part. They are very small, varying in size from a pin's head to a large lentil. Sometimes they are quite circular and as if clean cut out, but more often they are ellipsoid. Their edges are always thin and seldom detached, and it is rare to find them penetrating so deep as the muscular tissue. They much resemble superficial ulcers of the cornea in appearance. In almost all cases the bronchi suffer severe lesions also, but are not ulcerated. The entire number of ulcerations observed was 16; of which nine occurred in the larynx, four in the trachea, and three in both these parts. In one-half of these the disease remained latent, without furnishing any symptoms. In the other cases, change of voice and pain in the region of the larynx were observed. The ulcers are found generally in children more than seven years of age.

7. *Tuberculization of the Peritoneum* (*Peritoneal Phthisis*).

The various forms of tubercular deposition may take place in the peritoneum, but in different degrees of frequency. Thus, of 86 cases, the yellow granulation was found in 43, the grey in 24, miliary tubercle or tubercular patches in 37. The deposit was intra-serous in 40, extra-serous in 22, both of these in 22, and of doubtful seat in 10. Sometimes the deposition is enormously abundant, binding various organs to each other, but at others it only occupies very limited spots of the serous membrane. It was found abundant in 20 instances, moderately so in 14, and in small quantity in 42. The adhesions of the contiguous surfaces of the peritoneum, produced by the irritation of the sub-serous tubercles, present a great obstacle to perforation of the intestines. Tubercles which arise in the cavity of the serous membrane have no tendency to perforate, but those arising on its external surface possess such tendency.

*Symptoms.*—There are few of these which are sufficiently distinctive of the disease at its commencement, or even after it has progressed. The swelling of the belly which occurs is uniform and *ovoid*, accompanied with tension, and sometimes with indistinct fluctuation. It is generally sonorous, but in some cases only so in portions of its extent. After the disease has existed a certain time we observe the skin to become shining and desquamating, and sometimes the abdominal veins become dilated. In several children, from two to three years of age (when gaseous distension is very common), all these symptoms have existed, and yet the peritoneum has been found after death unaffected. Vomiting, which is so common in acute peritonitis, is absent here; while, on the contrary, purging is constantly present. The disease is often cut short by the supervention of some other lesion, but all periods of duration between one and seven months have been observed. Even skilful practitioners have frequently mistaken an anormal development of the abdomen for this disease, especially in ricketty children. But it should be remembered that peritoneal phthisis is very rare in *very young children*, the usual subjects of rickets, in whom too the abdomen is globular, soft, and without tension.

Peritonitis may become developed in tubercular children both when the peritoneum is, and when it is not, the seat of tubercle.

8. *Tuberculization of the Mesenteric Glands (Mesenteric Phthisis)*.

According to most authors this is one of the most frequent affections of infancy. It is far, however, from being so, and has been only thought so from the large belly of very young children having been mistaken for it, and peritoneal phthisis, intestinal ulceration, &c. having been confounded with it. If, indeed, we are to reckon all the cases as mesenteric disease in which slight affection of the glands exists, it is of very frequent occurrence, since this has been observed in one-half the tuberculous children (144). If we confine ourselves to those in whom it is considerable, there is but one seventh of the whole number of those who have mesenteric tubercles, and but one sixteenth of those in whom tuberculization exists in some part of the economy.

The mesenteric glands sometimes acquire an immense development when tuberculous, remaining crude or softened in part, but rarely forming cavities. The portion of gland surrounding the deposition occasionally remains healthy, and at others manifests inflammatory action. Considerable tuberculization of these glands ordinarily coincides with tuberculization of other organs, but the coincidence of mesenteric and peritoneal phthisis is rare. The disease is often difficult of determination, especially in its early stages, when the tubercle cannot be felt. The belly is seldom, at any period, swollen and tense, as in the peritoneal form. The tumours, which are always situated near the umbilicus, frequently cannot be detected, even when of considerable size. The voracity, said to be characteristic of the disease, is found in chronic peritonitis, and other tuberculizations. Purging was always present, but there also existed intestinal ulceration. So unattended with peculiar symptoms does the disease continue for some time, that its duration cannot be accurately ascertained. It is a vulgar error that this disease is one especially affecting young children; for it is hardly ever seen in its severe form under three years of age. The investigations of the authors lead them to the conclusions—(1) That the disease is slight in proportion as the child is young. (2) It is observed at its maximum of development between the 5th and 10th year. (3) It rarely occurs in any form between 12 and 15. Boys are more liable to the disease than girls. Of the *prognosis* we have the following remarks :—

"If this disease continued concentrated in the abdomen, its prognosis would not be so serious as that of the tuberculization of other organs, for the local accidents produced by the tumours are rare, and offer much less gravity than those of peritoneal or bronchial phthisis. We do not observe in *tabes mesenterica* either serous inflammation, the consequence of perforation, or intestinal hæmorrhage. The other symptoms are but slight; the wasting being less rapid, and the fever less marked, than in pulmonary or peritoneal phthisis. The gravity of the disease then does not depend upon the local affection, but upon the general malady, and especially upon the pulmonary complications."

### 9. *Gastro-Intestinal Tuberculization.*

The gastro-intestinal is the only mucous membrane in which tubercle has been detected. The miliary tubercle, either crude or softened, and, as its consequence, intestinal ulceration, is the only form of deposition that has been seen. It is especially found at the lower end of the small intestine. In 141 autopsies in which tubercle or ulcerations existed, these were found 21 times in the stomach; 134 in the small intestines; 60 in the large, occurring especially in the cœcum. Ulceration of the intestine, which is so common here, is, properly speaking, an epiphenomenon of general tuberculization, and accompanies also other forms of phthisis. It gives rise to a persistent diarrhœa, which is generally, but not always, proportioned to its extent, and is usually (as are the ulcers) most abundant and continuous in the older children. In 52 cases, in which the ulcers were few, the diarrhœa was yet as abundant, but then there were, in most of these, other secondary inflammatory affections present. In the other cases the diarrhœa was slight. All the children who did not suffer from diarrhœa were attacked with tubercular meningitis. Most of the children in whom ulcers existed, were likewise the subjects of tubercular peritonitis, or mesenteric phthisis. Prolonged diarrhœa is not of the same signification as indicating ulceration of the intestine in the child as in the adult, since it frequently arises from simple chronic enteritis.

The ramollissement of the gastro-intestinal membrane is a not unfrequent *secondary* lesion in phthisical children, especially such as have died of meningitis. It is revealed by no symptom, and frequently occurs shortly prior to death, or as a cadaveric change.

10. *Tuberculization of the Liver.*

This, which is rare in the adult, is not so in the child; for, of 312 tuberculous children, in 71 tubercles were found in the liver, the miliary and grey granulation being the forms usually observed. The deposit acquires a much deeper colour here than in any other organ. In 14 of the cases the tubercle was converted into small cavities or cysts, filled with a bilious-looking fluid.

*Fatty Degeneration of the Liver.*—This change in the liver of phthisical patients is less common in children than in adults, and is found as a sequence of other diseases also, especially the eruptive and typhoid fevers. In the 312 tuberculous children it has been observed 23 times, and in 211 non-tuberculous 14 times. It is very rare to find tubercle in a fatty liver. Very young children are more disposed to it than older ones, and especially when tuberculization is only slight.

11. *Tuberculization of the Kidneys.*

Instances of tuberculization of the kidney are much more frequently met with in the child than in the adult; "but it is rare to find it acquiring a high degree of intensity, or constituting important changes." All advanced cases recorded have occurred in children at least ten years of age, being then usually a part of an advanced general tuberculization. Of the 312 tuberculous patients, the kidneys were found affected 49 times, in 15 of which the deposit was pretty abundant, and in 34 only scanty; occurring in 37 instances in both kidneys. The disease is generally latent, and seems to exert but little influence upon the progress or termination of the general tuberculization.

12. *Tuberculization of the Spleen.*

This is one of the organs in which tubercle is deposited with most frequency and greatest abundance in childhood. Of the 312 cases, 107 presented tuberculous matter in the spleen, in 87 of which it was of the miliary form. When the tubercles are very abundant they may distend and enlarge the organ, but sometimes there is also hypertrophy independently of the tubercular deposit, which may then be a mere coincidence. There are no special symptoms. Boys and the youngest children are more subject to the disease than girls and older children.

13. *Tuberculization of the Brain and its Membranes.*

Tubercles may be deposited in the membranes or substance of the brain. The former are more frequent of occurrence and in number, and where the two co-exist it is often difficult to say in which site the deposition first took place. Generally of small size, tubercle, when seated in the cerebral substance, sometimes acquires large dimensions, exceeding those of the egg of a fowl.

MM. R. and B. go into considerable details as to the characters, symptoms, &c. of each of these affections, but our exhausted space forbids our following them. This is of the less consequence, since most of the symptoms incident upon deposition of tubercle in the brain (when the disease is not latent, as it sometimes is) are similar to those induced by other chronic diseases of that organ: while *Tuberculous Meningitis* being, in the opinion of the French school, identical with *Acute Hydrocephalus,* we shall have another opportunity of adverting to the facts adduced, in our review of Dr. Smith's work upon that subject.

It may be useful, in conclusion, to extract a Synopsis of the relative frequency of the occurrence of tubercle in the various organs of the 312 tuberculous children examined.

| | Total. | Considerable. | Medium. | Slight. |
|---|---|---|---|---|
| " Lungs .. .. .. .. .. | 265 | 71 | 52 | 142 |
| Bronchial Glands .. | 249 | 69 | 77 | 103 |
| Mesenteric Glands .. | 144 | 20 | 48 | 76 |
| Small Intestines.. .. | 134 | 50 | 14 | 70 |
| Pleura.. .. .. .. .. | 109 | 21 | 35 | 53 |
| Spleen.. .. .. .. .. | 107 | 25 | 25 | 57 |
| Peritoneum.. .. .. | 86 | 20 | 24 | 42 |
| Liver .. .. .. .. | 71 | 14 | 18 | 39 |
| Large Intestines.. .. | 60 | 10 | 18 | 32 |
| Membranes of Brain.. | 52 | 12 | 20 | 20 |
| Kidneys .. .. .. | 49 | 5 | 10 | 34 |
| Brain .. .. .. .. .. | 37 | 12 | 9 | 16 |
| Stomach .. . .. | 21 | 2 | 4 | 15 |
| Pericardium .. .. | 10 | 2 | 1 | 7 |

" One organ alone was tuberculous but in 48 instances, viz. in the Lungs 23 times; the Bronchial glands 19 times; the Pleura and Brain each twice: Membranes of the Brain and Kidneys each once."

The last class, *Intestinal Worms,* does not call for any notice at our hands; and, in terminating our analysis of this treatise of MM. Rilliet and Barthez, we need only repeat the statement of the great satisfaction we have derived from its perusal, and cordially recommend it to our readers. We sincerely hope that the same co-operative industry, patient investigation of truth, and impartial statement of results, may continue to be employed in other extensive and important fields of research.

